# Standardisation framework for the Maudsley staging method for treatment resistance in depression

**DOI:** 10.1186/s12888-018-1679-x

**Published:** 2018-04-11

**Authors:** Abebaw Fekadu, Jacek G. Donocik, Anthony J. Cleare

**Affiliations:** 1 Centre for Innovative Drug Development and Therapeutic Trials for Africa (CDT-Africa), College of Health Sciences, Addis Ababa University, Addis Ababa, Ethiopia; 2 Global Health & Infection Department, Brighton and Sussex Medical School, University of Sussex, Brighton, UK; 3 King’s College London, Institute of Psychiatry, Psychology and Neuroscience, Department of Psychological Medicine, Centre for Affective Disorders, London, UK

**Keywords:** Depressive disorder, Treatment-resistant depression, Remission, Staging; Maudsley staging method

## Abstract

**Background:**

Treatment-resistant depression (TRD) is a serious and relatively common clinical condition. Lack of consensus on defining and staging TRD remains one of the main barriers to understanding TRD and approaches to intervention. The Maudsley Staging Method (MSM) is the first multidimensional model developed to define and stage treatment-resistance in “unipolar depression”. The model is being used increasingly in treatment and epidemiological studies of TRD and has the potential to support consensus. Yet, standardised methods for rating the MSM have not been described adequately. The aim of this report is to present standardised approaches for rating or completing the MSM.

**Method:**

Based on the initial development of the MSM and a narrative review of the literature, the developers of the MSM provide explicit guidance on how the three dimensions of the MSM--treatment failure, severity of depressive episode and duration of depressive episode-- may be rated.

**Result:**

The core dimension of the MSM, treatment failure, may be assessed using the Maudsley Treatment Inventory (MTI), a new method developed for the purposes of completing the MSM. The MTI consists of a relatively comprehensive list of medications with options for rating doses and provisions treatment for multiple episodes. The second dimension, severity of symptoms, may be assessed using simple instruments such as the Clinical Global Impression, the Psychiatric Status Rating or checklist from a standard diagnostic checklist. The standardisation also provides a simple rating scale for scoring the third dimension, duration of depressive episode.

**Conclusion:**

The approaches provided should have clinical and research utility in staging TRD. However, in proposing this model, we are fully cognisant that until the pathophysiology of depression is better understood, staging methods can only be tentative approximations. Future developments should attempt to incorporate other biological/ pathophysiological dimensions for staging.

**Electronic supplementary material:**

The online version of this article (10.1186/s12888-018-1679-x) contains supplementary material, which is available to authorized users.

## Background

Treatment-resistance is a common clinical phenomenon in medicine. In chronic conditions like epilepsy, multiple sclerosis and hypertension, at least 30% of patients fail to respond adequately (attain remission of symptoms) to their first medication [1–3]. Ten to 15% tend to suffer chronically. Although depressive disorders may have a more favourable prognosis compared with primary psychotic disorders such as schizophrenia [4, 5], nevertheless 20%–40% of patients treated fail to respond to their first treatment with antidepressants [6] and up to 15% to multiple antidepressants [7].

The history of treatment-resistance in depression is as old as the history of antidepressant treatments itself. Soon after the introduction of imipramine [8], Robert Kuhn conceded that “in many cases, however, there is merely some degree of improvement, making the condition more bearable for the patient, and even permitting resumption of work, though at the cost of considerable effort. In other cases there is no effect at all” [8]. Kuhn’s observation was confirmed and quantified within few years, in two key studies, one from the United Kingdom (UK) and another from the United States (US). The US study compared the efficacy of imipramine or isocarboxazid and phenelzine against electroconvulsive therapy [9]. Overall, poor treatment response was observed in 50% of those receiving medication. The proportion for ECT was lower (25%) while that for isocarboxazid was the highest (66.7%). In a multi-centre study of the Medical Research Council (MRC) in the UK, involving 269 inpatients, 16% of those receiving ECT, 28% on imipramine and 62% on phenelezine failed to show improvement [10]. When a more strict definition of improvement was applied as having no or only “slight symptoms”, the rate failing to show improvement increased to 27% for ECT, 48% for imipramine and 70% for phenelezine. In 1974, the WHO convened a conference on TRD and attempted to define the concept, which led to a series of related publications [11–14]. Nearly 50 years later, despite the development of relatively diverse treatment options, and better understanding of optimisation strategies, at least 30% of people with depression do not show satisfactory improvement [15]. Thus treatment-resistance has remained a relatively common occurrence since the beginning of the psychopharmacology of depression and continues to be part of day to day clinical practice.

When treatment-resistance develops, the burden to patients comes not just from distressing symptoms and the associated disability but also from the treatment. Those who become treatment resistant often receive regimens combining two or more different medications, with potential longer-term side effect burden. Although it is difficult to disentangle the adverse effects on physical health of depression from that of medication use, there are suggestions that long-term use of moderate doses of antidepressants has been associated with the development of diabetes [16] and ischaemic heart disease [17, 18]. Also, as exemplified in depression, patients with treatment-resistant conditions are likely to suffer from co-morbid physical and mental disorders, to experience marked and protracted functional impairment, and to incur significantly higher healthcare costs [19–22]. All this underscore the public health relevance of TRD and the need for focused research into its aetiology, epidemiology and treatment. Although the first essential step to conduct such a research is to have a consensus in defining what constitutes treatment-resistance and to establish appropriate methods for staging its severity, so far there is no consensual definition or staging for treatment-resistance [23]. At the heart of the challenge in defining and staging TRD remains the lack of external validator or biological and physiological marker of depressive disorders and response to treatment. These markers will transform our approaches to staging treatment-resistance. Until then, helpful approaches to staging, including multi-dimensional models are being developed.

### The Maudsley staging method

To support the effort to better understand and stage TRD, we developed a multidimensional staging model, the Maudsley Staging Method (MSM) [24]. The initial development of the model was based on extensive literature review, and systematic assessment of the dimensions making the MSM as well as testing of the construct using original data. The MSM has shown promising predictive validity for both short-term [24–26] and longer-term outcomes [27, 28] of TRD. In addition to indications of construct validity based on more elaborate evaluation [29], the MSM has also been used for screening purposes in clinical trials [30, 31] and in studies of determinants of treatment outcomes [32].

The tool was developed as a loosely structured instrument such that a clinician with mental health training would be able to complete it. However, we have not published a detailed guidance on how the MSM should be completed. We have received many requests by researchers to provide such guidance to help standardise the completion of the MSM. The primary aim of this paper is to offer tools for standardisation of the MSM. The paper also provides context by providing an overview of the definitions and staging methods of treatment-resistance in depression and by providing an overview of the main staging methods to date.

## Methods

The methods were guided by three questions relevant to the objectives of the study: (1) How is TRD defined and what are the staging methods for TRD employed in clinical practice and research? (2) Are there any approaches or standardised methods being used to complete the staging strategies? (3) What are the recommended outcomes targets of treatment and how are these measured? We relied on three complementary approaches to answer these questions. (1) Methods used in the initial development of the MSM; (2) narrative review of the literature review; (3) review of treatment guidelines, such as the American Psychiatric Association’s practice guideline for the treatment of depression [33]; the British Association for Psychopharmacology guideline for the treatment of depression [34]; the Maudsley Prescribing Guideline [35]; and the depression treatment guideline of the World Federation of Societies of Biological Psychiatry [23, 36].

Narrative review was chosen because of the need to focus on high level answers to the questions raised above given the relatively broad nature of the questions asked. We were also convinced that a broad range of papers of sufficient quality would be obtained through this method. Nevertheless, we borrow some approaches from systematic review methodology to make sure all key works in the field of research are captured and minimise risk of bias. Thus, we searched in Embase, Medline and PsycInfo databases using key terms relevant for treatment resistant depression and staging developed in Pubmed. The search terms were depressive disorder, treatment-resistant or treatment-resistant depressive disorder combined using the Boolean ‘AND’ operator with staging methods. The reviewed literature was imported into Endnote software.

### Ethical considerations

Not applicable.

## Results

We begin by describing the definitions and staging of TRD and then provide specific tools for completing or rating the MSM. These tools include measures of the MSM dimensions, illness severity and remission, duration of illness and treatment. We provide a new instrument to collect data on the treatments offered during the course of illness, the Maudsley Treatment Inventory (MTI).

### Definitions

#### Definitions from treatment studies

A review of 47 treatment trials explored the definitions and staging criteria in TRD [37]. Lack of consensus was described in both the definition of the depressive syndrome and in how treatment response was operationalised. The depressive syndrome was defined either using rating scales (for example, the Hamilton Rating Scale for Depression (HRSD) [38]) or a standard operationalized diagnostic system, such as the Diagnostic and Statistical Manual of Mental Disorders (DSM) [39]. The required number of antidepressant failure to define treatment non-response was failure of at least one antidepressant medication in about a quarter of studies, while about half required non-response to at least two antidepressant medications [37]. Treatment non-response was characterized either in terms of failure to achieve a specified percentage reduction in the score of a rating scale or the continuation of a major depressive episode despite treatment. Most studies defined treatment failure only in relation to the presenting episode while few also included treatment failures, or recurrences whilst on treatment, in previous episodes. Commonly used terms for TRD included “difficult to treat” depression, “refractory” depression, “therapy-resistant” depression and “intractable” depression.

#### Definitions from treatment guidelines and staging methods

Several agencies have attempted to define treatment-resistance either directly or indirectly (Table 1). The British Association for Psychopharmacology (BAP) highlights that the definitions of treatment resistance vary and concludes that “most described it as a failure to respond to two or more adequate antidepressant treatment trials”. The authors acknowledge important problems with the definition that “arise in defining what comprises an adequate treatment trial, which drugs are to be included and in taking account of psychological treatments” [40]. The National Institute of Clinical Excellence (NICE) defined treatment-resistance in a similar way as failure to respond to two or more sequentially given antidepressant medications [41].

**Table 1:** The main definitions of treatment-resistance in depression

Year	Source	Definition	Remarks
1974	WHO	Failure of 150 mg of imipramine or equivalent given for 4 weeks	Specifies dose and duration Also specifies relative and absolute resistance based on dose of imipramine (150 mg being the threshold for absolute resistance)
1997	Thase & Rush	Failure of 1 adequately given antidepressant medication	Primarily for staging; Assumes hierarchy
1999	Sourey et al	Failure of 2 antidepressant medications from different classes	Also called the European method; failure of 1 antidepressant medication defined as non-response; uses chronicity criteria for staging
2002	EMEA	Failure of 2 medications from different classes	Suggests remission as outcome criteria
2000/ 2005	APA	Failure of 1 adequately given antidepressant medication given for 4–8 weeks	Not attempt to define directly but implicit reference can be interpreted
2003	MGH	Failure of 1 antidepressant medication?	Primarily staging method
2007	NICE	Failure of 2 antidepressant medication from different classes	
2008	BAP	Failure of 2 antidepressant medication from different classes	Refers to commonly used definitions instead of attempting to provide a definition of its own
2009	MSM	Failure of 1 adequately given antidepressant medication	Failure to achieve remission suggested as main outcome criteria; Primarily for staging
2015	DM-TRD	Failure of 1 adequately given antidepressant medication	Based on the MSM
2017	Conway et al	Failure of 2 adequate dose-duration antidepressants or psychotherapy from different classes	Antidepressants given in current episode. Combinations count individually

Abbreviations: APA = American Psychiatric Association; BAP=British Association of Psychopharmacology; DM-TRD = The Dutch Measure for quantification of Treatment Resistance in Depression (DM-TRD); EMEA = The European Agency for the Evaluation of Medicinal Products; MGH = Massachusetts General Hospital (staging method); MSM = Maudsley Staging Method; NICE = National Institute for Health and Clinical Excellence; WHO=World Health Organisation

(*References provided in main text*)

The European Agency for the Evaluation of Medicinal Products [42]—EMEA—comments that “a patient is considered therapy resistant when consecutive treatment with two products of different classes, used for sufficient length of time at an adequate dose, fail to induce an acceptable effect”. It further notes that the treatment “end point should be relevant to this patient group, e.g., remission may be more important than mean change in a scale.” It specifies the 17-item HRSD, the Montgomery Åsberg Depression Rating Scale, and the Clinical Global Impression scale as acceptable scales for use to determine symptomatic improvement.

The American Psychiatric Association (APA) [33] refers to “failure to respond” to treatment and interprets this to mean failure of exhibiting at least a moderate level of improvement following 4–8 weeks of pharmacotherapy or psychotherapy. It also specifies that following any change of treatment regimen, lack of improvement in the symptoms of major depressive disorder after an additional treatment period of 6–8 weeks would constitute failure of response. Unlike the definitions above, the APA definition implies that failure of one medication might be adequate to define treatment-resistance. This is more explicit in another related definition that uses the term “treatment-refractory”. “Refractory depression is defined as an episode of major depression, not secondary to a medical or drug-induced condition, which fails to respond (or to maintain a response) to an adequate trial of an antidepressant drug of established efficacy. An adequate trial is defined as 6 weeks of treatment with antidepressant at dosage considered therapeutic.” [43].

In the widely used staging method of TRD, the Thase and Rush model [44], failure to respond to a single adequately given antidepressant medication is implicitly indicated to constitute TRD [44]. Recent evidence indicates that failure of the first antidepressant treatment may be the gateway towards subsequent treatment failures, especially when the failure was not due to medication intolerance [15]. Similarly, the MSM, and a more recent multi-dimensional staging method (The Dutch Measure for quantification of Treatment Resistance in Depression (DM-TRD)) [45] specify failure of an adequately given antidepressant medication to be the core feature of TRD. The Medicare Evidence Development and Coverage Advisory Committee met in April 2016 but did not provide an explicit definition of what the threshold for TRD should be [46]. However, some participants of the meeting proposed that two failed medications or failure of an eight session psychotherapy would constitute a TRD [47].

### Current staging methods of TRD

Six staging methods were identified. The Thase and Rush model (TRM) [44] is the most widely used model. TRM offers a hierarchical model of staging [48] in which medications used at the higher order of treatment resistance are implicitly assumed to have superior efficacy. Despite the limited observational evidence, the TRM, by virtue of its hierarchical nature, implies that MAOIs may be of benefit in inducing and maintaining remission in TRD [27]. The hierarchical assumptions and limited flexibility to accommodate the potentially numerous medications that may be used to treat a resistant episode are the major drawbacks of the TRM. The hierarchical model also assumes that medication would be given in a certain sequence, progressing from relatively safe medication to the use of medications with potentially more serious side effects and culminating in the use of electroconvulsive therapy (ECT). However, in clinical practice, treatment is prescribed in an individualised way with informed negotiation rather than in a predetermined sequence in which ECT is the treatment of last resort. Furthermore, in current practice, much more stringent criteria [41, 49, 50] favour the use of ECT in life threatening emergencies. As discussed above, despite some suggestive reports [51] and the historical assumptions, there is also no robust evidence supporting the superiority of switching to a different antidepressant class as opposed to switching within class [15, 19, 52], as implied in the model. Neither are there clear provisions in the model for combination or augmentation strategies [19].

In the Massachusetts General Hospital staging method (MGH-S) [19], the staging of treatment resistance is mainly based on the number of antidepressant medications used. A special weight is given for failure of treatment with ECT, which receives a score equivalent to three antidepressant failures. There is some limited evidence on the utility of this model [53]. The model allows flexibility to incorporate as many failed treatment attempts as required; however, given the potential for a large number of treatment options available currently, the system may be less efficient and less discriminating. Thus data obtained may not inform intervention strategies or enhance understanding and communication. There is also no clear evidence supporting the magnitude of the special weight given to treatment with ECT.

A third method, which is sometimes called the European method of staging relies on matching treatment resistance to specific class of medication used and duration of treatment trials [54]. The model distinguishes treatment *non-response* from *treatment-resistance*. The former is when there is lack of response to one adequately used antidepressant medication; the latter is applied when two antidepressants fail. Based on the duration and intensity of treatment trial, this method classifies treatment-resistance into acute (TRD of less than 12 months) and chronic TRD. The acute subtype of TRD has five hierarchical categories. The first category, TRD 1, is assumed when medication trial of 12–16 weeks fails. The hierarchy is then built in what appears to be an ad hoc fashion, in which intervals of 4–36 weeks trial period divides the subsequent hierarchies or levels. Chronic resistant depression is diagnosed when patient fails to respond to several antidepressant medications in a treatment trial period that has lasted at least 12 months. Although the recognition of chronicity in this model is relevant, the cut off duration for chronicity (12 months) is not in line with previous recommendations [55] and diagnostic systems [39]. The model is also limited in scope, and its assumption regarding the differential effectiveness of antidepressant medications does not have clear supporting evidence.

A staging model based on depression subtypes on a dimension of severity (psychotic, melancholic and non-melancholic) [56] has been shown in a cross-sectional assessment to have convergent validity with clinician impression of resistance [56]. This model is parsimonious, which also makes it of narrower scope. Given the nature of the severity specifiers, this staging method may tap into bipolarity related treatment failure, which does not always represent true treatment failure.

In addition to what has been discussed above, the key shortcoming of these staging models is their reliance on a single criterion, mainly treatment response [44]. Although failure of antidepressant medication to induce improvement is the sine qua non of treatment-resistance, basing staging methods solely on medication use to the exclusion of other relevant factors such as duration and severity of illness, type of depression and the role of psychosocial stressors has been criticised [57].

**Fig. 1:**
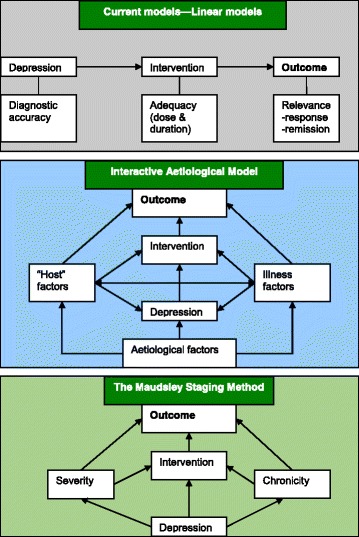
Current linear staging models (first figure) compare with the Maudsley staging method (last figure) and a more ideal interactive aetiological model (middle figure)

Considering these shortcomings in the available methods, two multi-dimensional scales have been developed [24, 45]. The Maudsley Staging Method (MSM), was developed by the authors of this paper. Its utility in predicting short- and medium-term outcome was also confirmed [24, 27, 28]. Although far inferior to an aetiological model, the MSM has improved potential compared with the traditional linear models of staging (Fig 1). The Dutch Measure for quantification of Treatment Resistance in Depression (DM-TRD) [45]. This method was developed from the MSM and extends the MSM by adding items for functional impairment, comorbid anxiety, personality disorders and psychosocial stressors. The DM-TRD also adds items for failed psychotherapy. The authors evaluate the inter−/intra-rater reliability and report ‘excellent’ reliability and good predictive validity.

### The Maudsley staging method: Considerations in developing the method

#### Definition of treatment-resistance

The MSM defines treatment-resistance as: *failure to attain significant level of improvement (equated with clinical remission) from an accurately defined depressive episode following treatment with an antidepressant medication given at an adequate (minimum effective) dose for a minimum of six weeks.* Given the role of the failure of the initial treatment as a gateway for treatment-resistance, the MSM uses failure of the first antidepressant treatment to designate the onset of treatment- resistance. Resistance is not an all-or-nothing phenomenon. It exists as a continuum and various factors (dimensions) contribute to its occurrence and maintenance. Three dimensions are considered (Tables 2 and 3). The first and the core dimension is treatment failure. The MSM incorporates severity (dimension 2) and duration of the depressive episode (dimension 3) as important dimensions to quantify treatment-resistance. These dimensions are expanded upon below because understanding the assumptions of the MSM are important for understanding and using the proposed rating approaches.

**Table 2:** Treatment dimension of the Maudsley Staging Method & suggested scoring conventions

Treatment	Categories	Scores
Treatment failures		
*Antidepressants*	Level 1: 1–2 medications	1
	Level 2: 3–4 medications	2
	Level 3: 5–6 medications	3
	Level 4: 7–10 medications	4
	Level 5: > 10 medications	5
*Augmentation*		
	Not used	0
	Used	1
*ECT*		
	Not used	0
	Used	1
Total maximum	–	(7)

**Table 3:** Illness dimensions of the Maudsley Staging Method & suggested scoring conventions

Parameter/dimension	Parameter categories	Score
Duration		
	Acute (≤12 months)	1
	Sub-acute (13–24 months)	2
	Chronic (> 24 months)	3
Symptom severity		
*(At baseline)*	Subsyndromal	1
	Syndromal	
	Mild	2
	Moderate	3
	Severe without psychosis	4
	Severe with psychosis	5
Highest score for illness dimensions	–	8
Overall maximum score for MSM		15
Severity score categories of the MSM	Severity range	3–15
	Mild	3–6
	Moderate	7–10
	Severe	11–15

#### Dimension 1-treatment failure

Failure of the first treatment appears important in that once the first treatment trial fails, the response rate to each successive treatment declines [15]. This implies that failure of the first treatment is a meaningful starting point in the measure of this conceptual continuum. There is dearth of robust evidence supporting the superiority of employing switching compared with augmentation strategies [3, 15] as well as the type of treatment used [44, 53], while number of treatments sequentially failing to produce improvement seem indicative of increasing treatment-resistance [53, 56] and thus form the basis of the MSM staging criterion (Table 2).

It is proposed that antidepressant treatment should only count if treatment was given for six weeks. However, it can rightly be argued that four weeks could be a useful time frame given the need for early detection of TRD and evidence from earlier treatment trials and indications from guidelines [33, 58]. This is reflected in the instrument to assess treatment history, the Maudsley Treatment Inventory (MTI) (Additional file 1), which also includes rating for a four week treatment trial to aid further research into this question. Augmentation strategies [35, 59] and eight sessions of ECT [60]are also rated.

Failure of treatment is equated with failure to achieve clinical remission. Clinical remission is generally a heterogeneous phenomenon. The two main questions regarding the definition of remission relate to the threshold of improvement and duration of this improvement that would be required to designate the clinical state as remission. Establishing the threshold of improvement in treatment studies has relied on a serial assessment using depression rating scales, often the HRSD. The typical consensus based scores that indicate remission, for example score of 7 or less in the HRSD [61], do not often represent return to complete wellness [62–66]. The level of impairment or difficulty not only depends on the score but also on the type of symptoms that are still unresolved [66]. Thus, defining clinical remission may require scales, such as the LIFE-chart [67] that establish remission more explicitly. For the purposes of the MSM, we have used mainly the LIFE-chart method. However, until more validated methods or scoring systems are developed, using the available rating systems is unavoidable. We do not advocate using functioning as a measure of treatment success for the MSM even though functioning has been advocated as an ideal treatment goal [68]. Functioning is difficult to measure and is affected by various contextual factors. In terms of duration threshold, the definition of remission has also relied on the assessment instrument used, which varies from 1 week for the HRSD to 1 month in some PSR ratings. For the purposes of the MSM, a clinical definition of remission requires a single measure over the time frame of the instrument being used for the assessment (usually 1 to 4 weeks). More stringent definition, as that within the DSM of two months in remission can be used where sustained remission is the chosen clinical outcome of interest.

#### Severity of depression

Inclusion of severity of depression as a staging criterion not only makes clinical sense, but severity of illness has also been consistently associated with non-response in numerous treatment [69–72] and follow-up studies [73–77]. Severity of symptoms is the best predictor of persistence of depressive symptoms [78] and occurrence of residual symptoms and relapse [79, 80]. The association of severity of illness with outcome has been demonstrated for both severity determined by diagnosis according to specified criterion [81, 82] or measured by dimensional scales, such as the HRS-D [73].

The MSM was developed using the severity categories of the Mental and Behavioral Disorders section of the 10th revision of the International Classification of Diseases (ICD-10) [83]. Despite some uncertainties as to whether depression with psychotic symptoms may be a distinct disorder [84–86], we have included it as the most severe form of depression as is presented in both ICD-10 and DSM-IV. It is worth noting that the principle has continued in the new edition of the DSM, DSM-5 [87]. Utility of including subsyndromal severity level is demonstrated by the association of this level of depression with disability [88], poor quality of life [89] and relapse [88, 90]. Other approaches for estimating severity may be used (Table 4) but require further work. Although it is clear now that the current cut-off points for remission based on standard rating scales, such as the HRSD, do not correlate very well with functional recovery and satisfaction [63, 64, 66, 91], lower scores may not be pragmatic targets.

**Table 4:** Severity ratings compatible with the MSM for commonly used rating scales

Instrument	Clinical status categories based on severity scores						Remark
	Remission	Subthreshold	Mild	Moderate	Severe	Very severe	
QIDS-C16	0–5		6–10	11–15	16–20	> 20	Remission scores are likely to be too high and include subthreshold status
QIDS-SR16	0–5		6–10	11–15	16–20	> 20	
IDS-C	0–11		12–23	24–36	37–46	> 46	
IDS-SR	0–13		14–25	26–38	39–48	> 48	
HRSD17	0–7		8–13	14–19	20–25	26–52	
MADRS	0–6		7–19	20–34	35–60		
CGI	1	2	3	4	5–6	7	
PSR	1–2	3	4	5	6	7	

Abbreviations: CGI-Clinical Global Impression; HRSD17-Hamilton Rating Scale for Depression (17 item scale); IDS-C-Inventory of Depressive Symptoamtology, Clinician Rating; IDS-SR-Inventory of Depressive Symptomatology, Self-Report; MADRS-Montgomery-Asberg Depression Rating Scale*; PSR-Psychiatric Status Rating; QIDS-C-Quick Inventory of Depressive Symptomatology Clinician rated version; QIDS-SR-Quick Inventory of Depressive Symptomatology Self Rated version

Note: Adapted from http://www.ids-qids.org/interpretation.html (accessed on 29 Jan 2016) and the original sources of the instruments except for MADRS [108]

#### Chronicity

Studies have consistently demonstrated that the longer the duration of illness, the poorer the response to treatment in the acute phase of [81, 82, 92, 93] or augmentation [94] and predicted shorter relapse free survival [79, 95]. We based our model on the duration of the presenting depressive episode, irrespective of treatment experience. We classified duration into three categories. Duration of a year and less was considered acute, between one and two years as subacute and anything longer than two years as chronic (Table 3). The cut-off of two years for chronic depression was based on the criterion of the DSM-IV Text Revision (DSM-IV-TR) diagnostic system [96].

### How to complete (rate) the MSM: The MSM completion tool

The recommendations here target research settings where a standardised and replicable assessment is essential. This recommendation would also improve the utility of the MSM in specialist tertiary services, where patients with more complex needs and multiple treatment trials and treatment failures are seen. The tool was also developed with the clinical practitioner in mind. Clinicians can still continue to complete the MSM relatively quickly using the usual clinical history and benefit from the information for establishing baseline severity of treatment resistance as well as periodic monitoring.

### Rating for treatment failure (dimension 1)

We propose the use of one of three options to rate for treatment failure: The Antidepressant Response Questionnaire (ATRQ); The Antidepressant Treatment History Form (ATHF); or The Madusley Treatment Inventory (MTI). The MTI is a novel approach with multiple options for rating medication history. The MTI is described in detail at the end of this section.

#### The Massachusetts General Hospital (MGH) antidepressant treatment response questionnaire (ATRQ)

Is a self-rated instrument and defines adequate treatment trial as treatment at adequate doses of antidepressants for a duration of 6 weeks. The system provides operational criteria for adequacy of dosage for each of the most commonly used antidepressants. The strength of the ATRQ is the self-rated nature of the scale, which.

allows patients to indicate the level of benefit they feel they gained from the treatment. This is important because patients’ experience is the key outcome indicator. On the other hand, the subjective rating may be influenced by mood state of the person rating the instrument. Higher scores in self-rated scores (compared with observer-rated scales) may be reflection of personality factors [97]. Nevertheless, rating medications may be complex for patients and is important to have additional sources of information, such as clinical records, collateral information and other sources.

### The antidepressant treatment history form (ATHF)

Is a semi-structured tool that is used to define treatment resistance and treatment history (for current and past episodes), including somatic therapies [98–100]. It requires detailed information from different sources about the treatments and, for some medications, has provision for adequacy based on blood levels.

### The Maudsley treatment inventory (MTI)

The MTI is a semi-structured instrument that we have developed to document psychotropic medications and physical therapies used in the treatment of depression and assist the completion of the MSM. The MTI was developed from existing resources, mainly the Maudsley Prescribing Guideline [101], the BAP guideline [34] and the APA guideline [33]. The MTI is more comprehensive and potentially more suitable for rating TRD compared with other schedules developed to document treatment history.

The MTI should be completed using all available information-history from patient and care givers, clinical records as well as other sources, for example, results of structured evaluations. The inventory is primarily designed for use in the current episode, for which treatment resistance is being rated for. However, the MTI may also be used for rating treatment resistance for multiple episodes. If rating for multiple episodes, multiple MTIs need to be completed. The MTI lists medications available in the UK, but can be modified for use in other countries, by adding the new list of drugs available in the specific setting or modifying the brand names as appropriate. Preliminary assessment of “pseudo-resistance” can be made by evaluating treatment adherence, tolerability of treatment, and treatment response included in the MTI. “Pseudo-resistance” refers to an apparent treatment resistance in the face of misdiagnosis, inadequate treatment because of poor tolerability and poor adherence. For example, if a person was non-adherent for a substantial period of the follow-up time or was unable to tolerate a medication at an acceptable minimum effective dose, true treatment resistance is unlikely.

For the purposes of the MSM, we recommend using remission as the desired treatment outcome. Rating of treatment response can use the MTI response ratings or standard scale based ratings although we recommend the latter (Table 4).

### Dimension 2: Severity

The MSM was developed using an enhanced ICD-10 severity rating. This rating can be made using an ICD-10 symptom checklist. Equally acceptable would be to use a DSM 5 checklist. The main advantage of using the ICD/DSM for severity assessment is its clinical utility. However, the common approach in research and tertiary care settings is the use of standard severity rating instruments. Therefore, we recommend using standard instruments such as the HRSD and the QIDS whenever possible. Ratings for the five severity levels are proposed (Table 4). We are aware that the presence of even limited number of symptoms compatible with previous recommendations of remission would be associated with impaired functioning and quality of life [62, 64, 65, 91, 102, 103]. Nevertheless, at present, no concrete research data exists to provide cut-off scores in line with these recent findings. The only instruments that may allow clinical judgment about subthreshold symptoms and remission are the LIFE chart and the CGI. We have therefore taken the pragmatic approach and restricted our recommendations to what has been well established while awaiting further research.

### Dimension 3: Duration of depressive episode

The two key questions regarding duration of the depressive episode are: when should the onset of the depressive episode be dated? And what should the period of remission be to separate two apparently distinct episodes into two? In relation to dating onset, we propose provision of separate options for a new episode and relapse episode. For first episode, we propose dating the onset to the time of clear onset of a full episode of illness. For subsequent episodes, we propose to date the onset to the time when prodromal symptoms of relapse have begun. This distinction is made for the simple pragmatic reason that we know more about the contribution of subthreshold symptoms in relapse and maintenance of depressive episodes. However, relevance of these propositions has to be tested. The standard duration of remission to separate two episodes is two months. There is no clear reason as to why this duration was chosen, other than the assumption that, in the event of a new episode when the remission has been under two months, may simply be a continuation of the initial illness process rather than emergence of a relapse episode. There is clear uncertainty regarding duration of remission that heralds the onset of a more sustained remission. Further research in this area is warranted.

Three duration categories are recognised in the MSM. Rating these simply requires a standard clinical interview (Table 5), which enables accurate dating of the onset of the treatment-resistant episode. This should include the period prior to the initiation of the treatment.

**Table 5:** Rating for duration of depressive episode

Duration of depressive episode		
Duration Category	Duration	Rating
Acute	< 1 year	
Subacute	1 to < 2 years	
Chronic	2 years and above	

### Who should complete the MSM?

In our research and clinical practice at a tertiary centre, the MSM is completed by research or clinical psychiatrists and trainee psychiatrists. However, the MSM may be rated by a trained research nurse or junior research staff who can complete standard instruments, such as the HRSD. In research context where multiple users are likely to be involved, inter-rater agreement needs to be established.

## Discussion

Agreement on the definition of TRD has remained elusive in four decades. The inability of the recent Medicare Evidence Development and Coverage Advisory Committee to reach a consensus on defining TRD confirms the challenge. Nevertheless, treatment resistance is a vexed concept even in other chronic conditions. For example, an extensive review by the US Health Technology Assessment Group looking at literature spanning nearly 30 years and with the inclusion of 357 articles, failed to find consensus in the definition of treatment-resistant epilepsy (TRE) [104]. TRE was defined in less than a third of the studies. When a definition was given, it typically included the number of failed antiepileptic medications tried, and in some cases included the adequacy of dosage, the frequency of seizures and the duration of illness [104]. The authors also commented that “terms such as “intractable,” “refractory,” or “treatment-resistant” (*were used*)^1^ to describe patients for whom one or more treatments have failed, (*but*)^†^ no consensus exists as to precisely what these terms mean”. The expert panel then defined treatment-resistance as “failure of one or more antiepileptic drugs at a maximum tolerable dose to provide complete seizure relief”. In line with this consensus definition for RTE, and evidence that failure of the first antidepressant may be associated with subsequent reduced responsiveness, we have suggested that failure of one treatment should be the threshold for defining TRD. We thus suggest that failure to respond to the first treatment should count towards defining TRD. We also suggest that, despite its common usage, the term ‘refractory’ depression is a term that should no longer be used unless an end stage treatment-resistance is considered in which psychosurgery is being considered. The term refractory implies that virtually all chances of the person responding to treatment are gone. This proposition is contrary to reports of outcome studies, which suggest that, despite chronicity, most patients improve in the longer term with or without treatment [27, 105–107]. The term “refractory” may also have unwarranted neuro-physiological overtone, as in nerve conduction. There is no clear evidence to support the occurrence of a similar phenomenon in the treatment of depression. It therefore appears that the term “refractory” depression is at best poorly validated concept and at worst therapeutically unhelpful and can potentially encourage therapeutic nihilism. We suggest to no longer use this term until firm evidence confirms its validity, or use it only for a subgroup of patients with an agreed “end-stage” pattern of resistance.

Treatment-resistance is not an all or none phenomenon but is rather a continuum, and the preferable representation of treatment resistance would be to describe the level of treatment-resistance in terms of various severity grades. Such severity gradation would be useful as the term TRD itself is non-specific and generic.

Although remission remains a recommended treatment target, this is not always achievable and should not be a cause for therapeutic nihilism. Improving symptoms with antidepressants when the severity scores go down to the mild and subthreshold range may be even more challenging and the risk-benefit balance of psychotropic medications more difficult to determine. In the language of the BAP guideline for treatment of depression [34], treatment of treatment-resistant conditions should be guided by “pragmatism and clinical judgement” based on “the risk–benefit balance in specific situations rather than using an arbitrary cut-off. This requires taking into account an individual’s history and the availability of alternative evidence-based treatments…” Managing treatment-resistant mental illness is an “art of the possible”. Systematic follow-up and monitoring of patients without changing or adding medications may be of meaningful benefit to patients with treatment-resistant illness.

## Conclusions

The framework and tools of the MSM offer a platform for shared understanding and replicable research in TRD. However, further short- and long-term development work is required. For example, the definitions of remission, the threshold for TRD and how previous history of non-response to treatment should be incorporated into a staging method is unclear. The potential role of suicidality as severity indicator may need further exploration. Most importantly, the treatment implication of the MSM should be explored. Matching the staging with recovery goals of patients is another key challenge that requires further work.

## Additional file

Additional file 1: Maudsley Treatment Inventory – MTI. (DOC 348 kb)

## Abbreviations

APA: American Psychiatric Association
ATHF: The Antidepressant Treatment History Form
ATRQ: Antidepressant Response Questionnaire
BAP: British Association for Psychopharmacology
CDT: Africa-Centre for Innovative Drug Development and Therapeutic Trials for Africa
CGI: Clinical Global Impression
DSM: Diagnostic and Statistical Manual
DSM-IV-TR: DSM-IV Text Revision
ECT: Electroconvulsive Therapy
EMEA: European Agency for the Evaluation of Medicinal Products
HRSD: Hamilton Rating Scale for Depression
ICD: International Classification of Diseases
LIFE: Longitudinal Interval Follow up Evaluation
MADRS: Montgomery-Asberg Depression Rating Scale
MAOI: Monoamine Oxidase Inhibitors
MGH-S: Massachusetts General Hospital staging method
MRC: Medical Research Council
MSM: Maudsley Staging Method
MTI: Maudsley Treatment Inventory
NICE: National Institute of Clinical Excellence
PSR: Psychiatric Status Rating
QIDS: Quick Inventory of Depressive Symptomatology
TRD: Treatment Resistant Depression
TRE: Treatment Resistant Epilepsy
TRM: Thase and Rush model
UK: United Kingdom
US: United States

## Footnotes

1 Italicised words in bracket are added by authors for clarity of reading
