# Supplementary material for: Standardisation framework for the Maudsley staging method for treatment resistance in depression
Source: BMC Psychiatry. 2018 Apr 11;18:100. doi: 10.1186/s12888-018-1679-x (PMC5896127; doi:10.1186/s12888-018-1679-x)
Supplement: Supplementary file 1 — Maudsley Treatment Inventory – MTI. (DOC 348 kb) [file 12888_2018_1679_MOESM1_ESM.doc]

Additional File 1

| **Generic Name** | **Brand Names**  (UK, unless stated) | **Dose** | **Duration**  In weeks on a dose equal or greater than the minimum dose | **Tolerability & Response** | | | **Drug taken for at least:** | | | **Minimum Dose***  (mg/day unless stated) | **Equal or greater** | **Maximum**  **Dose***  (mg/day unless stated) | **Equal or greater** | **Augmentation** |
| --- | --- | --- | --- | --- | --- | --- | --- | --- | --- | --- | --- | --- | --- | --- |
|  |  |  |  | **Tolerability^a^** | **Adherence^b^** | **Response^c^** | **4 weeks** | **6 weeks** | **8 weeks** |  |  |  |  |  |
| **SECTION A: RECOGNISED ANTIDEPRESSANTS** | | | | | | | | | | | | | | |
| **Selective Serotonin Reuptake Inhibitors (SSRI)** | | | | | | | | | | | | | | |
| Citalopram | Cipramil |  |  |  |  |  |  |  |  | 20 |  | 40 |  |  |
| Escitalopram | Cipralex |  |  |  |  |  |  |  |  | 10 |  | 20 |  |  |
| Fluoxetine | Prozac |  |  |  |  |  |  |  |  | 20 |  | 60 |  |  |
| Fluvoxamine | Faverin |  |  |  |  |  |  |  |  | 100 |  | 300 |  |  |
| Paroxetine | Seroxat |  |  |  |  |  |  |  |  | 20 |  | 50 |  |  |
| Sertraline | Lustral |  |  |  |  |  |  |  |  | 50 |  | 200 |  |  |
| **Tricyclic Antidepressants (TCA)** | | | | | | | | | | | | | | |
| Amitriptyline | Tryptizol |  |  |  |  |  |  |  |  | 125 |  | 200 |  |  |
| Amoxapine |  |  |  |  |  |  |  |  |  | 200 |  | 300/600^H^ |  |  |
| Clomipramine | Anafranil |  |  |  |  |  |  |  |  | 125 |  | 250 |  |  |
| Desipramine |  |  |  |  |  |  |  |  |  | 125 |  | 250 |  |  |
| Dosulepin / Dothiepin | Prothiaden |  |  |  |  |  |  |  |  | 125 |  | 225 |  |  |
| Doxepin | Sinepin |  |  |  |  |  |  |  |  | 125 |  | 300 |  |  |
| Imipramine | Tofranil |  |  |  |  |  |  |  |  | 125 |  | 300 |  |  |
| Lofepramine | Gamanil / Lomont |  |  |  |  |  |  |  |  | 140 |  | 210 |  |  |
| Nortriptyline | Allegron |  |  |  |  |  |  |  |  | 75 |  | 150 |  |  |
| Protriptyline |  |  |  |  |  |  |  |  |  | 30 |  | 60 |  |  |
| Tianeptine | Stablon (US) |  |  |  |  |  |  |  |  | 25 |  | 50 |  |  |
| Trimipramine | Surmontil |  |  |  |  |  |  |  |  | 125 |  | 300 |  |  |
| **Monoamine Oxidase Inhibitors (MAOI)** | | | | | | | | | | | | | | |
| Isocarboxazid |  |  |  |  |  |  |  |  |  | 30 |  | 60 |  |  |
| Phenelzine | Nardil |  |  |  |  |  |  |  |  | 45 |  | 60/90^H^ |  |  |
| Tranylcypromine | Parnate |  |  |  |  |  |  |  |  | 20 |  | - |  |  |
| Moclobemide | Manerix |  |  |  |  |  |  |  |  | 300 |  | 600 |  |  |
| Selegeline | Eldepryl/Zelapar |  |  |  |  |  |  |  |  | 6 |  | 12 |  |  |

| **Generic Name** | **Brand Names**  (UK, unless stated) | **Dose** | **Duration**  In weeks on a dose equal or greater than the minimum dose | **Tolerability & Response** | | | **Drug taken for at least:** | | | **Minimum Dose**  (mg/day unless stated) | **Equal or greater** | **Maximum**  **Dose***  (mg/day unless stated)) | **Equal or greater** | **Augmentation** |
| --- | --- | --- | --- | --- | --- | --- | --- | --- | --- | --- | --- | --- | --- | --- |
|  |  |  |  | **Tolerability^a^** | **Adherence^b^** | **Response^c^** | **4 weeks** | **6 weeks** | **8 weeks** |  |  |  |  |  |
|  | | | | | | | | | | | | | | |
| **Tetracyclic Antidepressants (and related)** | | | | | | | | | | | | | | |
| Maprotiline | Ludiomil |  |  |  |  |  |  |  |  | 75 |  | 150/225^H^ |  |  |
| Mianserin | Bolvidon, Norval |  |  |  |  |  |  |  |  | 30 |  | 90 |  |  |
| Mirtazepine | Zispin |  |  |  |  |  |  |  |  | 30 |  | 45 |  |  |
| Nefazadone | Serzone |  |  |  |  |  |  |  |  | 200 |  | 600 |  |  |
| Trazodone | Molipaxin |  |  |  |  |  |  |  |  | 150 |  | 600 |  |  |
| **Serotonin-Noradrenaline Reuptake Inhibitors (SNRI)** | | | | | | | | | | | | | | |
| Desvenlafaxine | Pristiq (US) |  |  |  |  |  |  |  |  | 50 |  | 400 |  |  |
| Duloxetine | Cymbalta |  |  |  |  |  |  |  |  | 60 |  | 120 |  |  |
| Levomilnacipran | Fetzima (US) |  |  |  |  |  |  |  |  | 40 |  | 120 |  |  |
| Milnacipran | Ixel / Savella (EU / US) |  |  |  |  |  |  |  |  | 100 |  | 200 |  |  |
| Venlafaxine | Efexor |  |  |  |  |  |  |  |  | 75 |  | 375 |  |  |
| **Serotonin Modulators & Stimulators** | | | | | | | | | | | | | | |
| Vilazodone | Viibryd (US) |  |  |  |  |  |  |  |  | 10 |  | 40 |  |  |
| Vortioxetine | Brintellix |  |  |  |  |  |  |  |  | 10 |  | 20 |  |  |
| **Noradrenaline Reuptake Inhibitors (NRI)** | | | | | | | | | | | | | | |
| Reboxetine | Edronax |  |  |  |  |  |  |  |  | 8 |  | 12 |  |  |
| Viloxazine | Vivalan (US) |  |  |  |  |  |  |  |  | 200 |  | 600 |  |  |
| **Noradrenaline &Dopamine Reuptake Inhibitors (NDRI)** | | | | | | | | | | | | | | |
| Bupropion | Zyban |  |  |  |  |  |  |  |  | 300 |  | 400 |  |  |
| **Melatonin Agonists** | | | | | | | | | | | | | | |
| Agomelatine | Valdoxan |  |  |  |  |  |  |  |  | 25 |  | 50 |  |  |
| **Other** | | | | | | | | | | | | | | |
| Flupentixol | Fluanxol |  |  |  |  |  |  |  |  | 1 |  | 3 |  |  |

| **Generic Name** | **Brand Names**  (UK, unless stated) | | **Dose** | | **Duration**  In weeks on a dose equal or greater than the minimum dose | | **Tolerability & Response** | | | | | | **Drug taken for at least:** | | | | | | **Minimum Dose**  (mg/day unless stated) | | **Equal or greater** | | **Maximum**  **Dose***  (mg/day unless stated) | | **Equal or greater** | | **Augmentation** |
| --- | --- | --- | --- | --- | --- | --- | --- | --- | --- | --- | --- | --- | --- | --- | --- | --- | --- | --- | --- | --- | --- | --- | --- | --- | --- | --- | --- |
|  |  |  |  |  |  |  | **Tolerability^a^** | | **Adherence^b^** | | **Response^c^** | | **4 weeks** | | **6 weeks** | | **8 weeks** | |  |  |  |  |  |  |  |  |  |
| **SECTION B: RECOGNISED AUGMENTATION THERAPIES^†^** | | | | | | | | | | | | | | | | | | | | | | | | | | | |
| **First and Second Line Therapies** | | | | | | | | | | | | | | | | | | | | | | | | | | | |
| Aripiprazole | Abilify | |  | |  | |  | |  | |  | |  | |  | |  | | 2.5 | |  | | 20 | |  | |  |
| Olanzapine | Zyprexa | |  | |  | |  | |  | |  | |  | |  | |  | | 10 | |  | | - | |  | |  |
| Quetiapine | Seroquel | |  | |  | |  | |  | |  | |  | |  | |  | | 150 | |  | | 300 | |  | |  |
| Risperidone | Risperdal | |  | |  | |  | |  | |  | |  | |  | |  | | 0.5 | |  | | 3 | |  | |  |
| Lamotrigine | Lamictal | |  | |  | |  | |  | |  | |  | |  | |  | | 200 | |  | | 400 | |  | |  |
| Lithium | Camcolit / Priadel | |  | |  | |  | |  | |  | |  | |  | |  | | 0.4mmol/l | |  | | 1.2mmol/l | |  | |  |
| Liothyronine; tri-iodothyronine (T3) | Tertroxin | |  | |  | |  | |  | |  | |  | |  | |  | | 20ug | |  | | 50ug | |  | |  |
| Buspirone | Buspar | |  | |  | |  | |  | |  | |  | |  | |  | | 15 | |  | | 60 | |  | |  |
|  | | | | | | | | | | | | | | | | | | | | | | | | | | | |
| **Third Line Therapies** | | | | | | | | | | | | | | | | | | | | | | | | | | | |
| Amantadine | Symmetrel | |  | |  | |  | |  | |  | |  | |  | |  | | - | |  | | 300 | |  | |  |
| Cabergoline | Dostinex / Cabaser | |  | |  | |  | |  | |  | |  | |  | |  | | 2 | |  | | 2 | |  | |  |
| d-cycloserine |  | |  | |  | |  | |  | |  | |  | |  | |  | | 1000 | |  | | 1000 | |  | |  |
| Dexamethasone |  | |  | |  | |  | |  | |  | |  | |  | |  | | 3 | |  | | 4 | |  | |  |
| Dexamphetamine | Dexedrine | |  | |  | |  | |  | |  | |  | |  | |  | | 2.5 | |  | | 40 | |  | |  |
| Hyoscine | Buscopan / Kwells | |  | |  | |  | |  | |  | |  | |  | |  | | - | |  | | - | |  | |  |
| Ketoconazole | Nizoral | |  | |  | |  | |  | |  | |  | |  | |  | | 400 | |  | | 800 | |  | |  |
| Mecamylamine |  | |  | |  | |  | |  | |  | |  | |  | |  | | - | |  | | 10 | |  | |  |
| Methylphenidate | Ritalin/Concerta | |  | |  | |  | |  | |  | |  | |  | |  | | 20 | |  | | 40 | |  | |  |
| Modafinil | Provigil | |  | |  | |  | |  | |  | |  | |  | |  | | 100 | |  | | 400 | |  | |  |
| Nemifitide |  | |  | |  | |  | |  | |  | |  | |  | |  | | 40 SC | |  | | 240 SC | |  | |  |
| Oestrogen |  | |  | |  | |  | |  | |  | |  | |  | |  | | - | |  | | - | |  | |  |
| Omega 3 triglycerides |  | |  | |  | |  | |  | |  | |  | |  | |  | | 1000 | |  | | 2000 | |  | |  |
| Pindolol | Visken | |  | |  | |  | |  | |  | |  | |  | |  | | 7.5 | |  | | 15 | |  | |  |
| Pramipexole | Mirapexin | |  | |  | |  | |  | |  | |  | |  | |  | | 0.125 | |  | | 5 | |  | |  |
|  | | | | | | | | | | | | | | | | | | | | | | | | | | | |
| **Generic Name** | | **Brand Names**  (UK, unless stated) | | **Dose** | | **Duration**  In weeks on a dose equal or greater than the minimum dose | | **Tolerability & Response** | | | | | | **Drug taken for at least:** | | | | | | **Minimum Dose**  (mg/day unless stated) | | **Equal or greater** | | **Maximum**  **Dose***  (mg/day unless stated)) | | **Equal or greater** | **Augmentation** |
|  |  |  |  |  |  |  |  | **Tolerability^a^** | | **Adherence^b^** | | **Response^c^** | | **4 weeks** | | **6 weeks** | | **8 weeks** | |  |  |  |  |  |  |  |  |
|  | | | | | | | | | | | | | | | | | | | | | | | | | | | |
| **Third line Therapies (continued)** | | | | | | | | | | | | | | | | | | | | | | | | | | | |
| Riluzole | Rilutek | |  | |  | |  | |  | |  | |  | |  | |  | | 100 | |  | | 200 | |  | |  |
| S-adenosyl-L-methionine |  | |  | |  | |  | |  | |  | |  | |  | |  | | 400 IM | |  | | 1200PO | |  | |  |
| Testosterone |  | |  | |  | |  | |  | |  | |  | |  | |  | | - | |  | | - | |  | |  |
| Tryptophan |  | |  | |  | |  | |  | |  | |  | |  | |  | | 6000 | |  | | 9000 | |  | |  |
| Zinc |  | |  | |  | |  | |  | |  | |  | |  | |  | | 25 | |  | | 25 | |  | |  |
| Ziprasidone | Geodon / Zeldox | |  | |  | |  | |  | |  | |  | |  | |  | | - | |  | | 160 | |  | |  |
|  | | | | | | | | | | | | | | | | | | | | | | | | | | | |

| **Generic Name** | **Brand Names**  (UK, unless stated) | **Dose** | **Duration**  In weeks on a dose equal or greater than the minimum dose | **Tolerability & Response** | | | **Drug taken for at least:** | | | **Minimum Dose**  (mg/day unless stated)) | **Equal or greater** | **Maximum**  **Dose***  (mg/day unless stated) | **Equal or greater** | **Augmentation** |
| --- | --- | --- | --- | --- | --- | --- | --- | --- | --- | --- | --- | --- | --- | --- |
|  |  |  |  | **Tolerability^a^** | **Adherence^b^** | **Response^c^** | **4 weeks** | **6 weeks** | **8 weeks** |  |  |  |  |  |
| **OPTIONAL SECTION C: OTHER ANTIPSYCHOTICS AND MOOD STABILISERS**  **Atypical Antipsychotics** | | | | | | | | | | | | | | |
| Amisulpiride | Solian |  |  |  |  |  |  |  |  | - |  | - |  |  |
| Asenapine | Sycrest |  |  |  |  |  |  |  |  |  |  | 20 |  |  |
| Clozapine | Clozaril |  |  |  |  |  |  |  |  | - |  | - |  |  |
| Iloperidone | Fanapt (US / EU) |  |  |  |  |  |  |  |  | 2 |  | 24 |  |  |
| Lurasidone | Latuda |  |  |  |  |  |  |  |  | 40 |  | 80 |  |  |
| Melperone |  |  |  |  |  |  |  |  |  | - |  | - |  |  |
| Paliperidone | Invega |  |  |  |  |  |  |  |  | - |  | - |  |  |
| Sertindole | Serdolect |  |  |  |  |  |  |  |  | - |  | - |  |  |
| Zotepine | Zoleptil |  |  |  |  |  |  |  |  | - |  | - |  |  |
| **Typical Antipsychotics** | | | | | | | | | | | | | | |
| Chlorpromazine | Largactil |  |  |  |  |  |  |  |  | - |  | - |  |  |
| Fluphenazine | Modecate |  |  |  |  |  |  |  |  | - |  | - |  |  |
| Haloperidol | Haldol |  |  |  |  |  |  |  |  | - |  | - |  |  |
| Levomepromazine | Nozinan |  |  |  |  |  |  |  |  | - |  | - |  |  |
| Pericyazine | Neulactil |  |  |  |  |  |  |  |  | - |  | - |  |  |
| Perphenazine | Fentazine |  |  |  |  |  |  |  |  | - |  | - |  |  |
| Pimozide | Orap |  |  |  |  |  |  |  |  | - |  | - |  |  |
| Prochlorperazine | Prochlorperazine |  |  |  |  |  |  |  |  | - |  | - |  |  |
| Promazine | Sparine |  |  |  |  |  |  |  |  | - |  | - |  |  |
| Sulpiride | Sulpor/dolmatil |  |  |  |  |  |  |  |  | - |  | - |  |  |
| Thioridazine | Melleril |  |  |  |  |  |  |  |  | - |  | - |  |  |
| Trifluoperazine | Stelazine |  |  |  |  |  |  |  |  | - |  | - |  |  |
| Zuclopenthixol | Clopixol |  |  |  |  |  |  |  |  | - |  | - |  |  |
| **Anticonvulsants** | | | | | | | | | | | | | | |
| Carbamazepine | Tegretol |  |  |  |  |  |  |  |  | 600 |  | 1600 |  |  |
| Gabapentin | Neurontin |  |  |  |  |  |  |  |  | - |  | 2400 |  |  |
| Levetiracetam | Keppra |  |  |  |  |  |  |  |  | - |  | 4000 |  |  |
| Oxcarbazepine | Trileptal |  |  |  |  |  |  |  |  | 300 |  | 3000 |  |  |
| Phenytoin |  |  |  |  |  |  |  |  |  | 300 |  | 400 |  |  |
|  |  |  |  |  |  |  |  |  |  |  |  |  |  |  |
| **Generic Name** | **Brand Names**  (UK, unless stated) | **Dose** | **Duration**  In weeks on a dose equal or greater than the minimum dose | **Tolerability & Response** | | | **Drug taken for at least:** | | | **Minimum Dose**  (mg/day unless stated) | **Equal or greater** | **Maximum**  **Dose***  (mg/day unless stated) | **Equal or greater** | **Augmentation** |
|  |  |  |  | **Tolerability^a^** | **Adherence^b^** | **Response^c^** | **4 weeks** | **6 weeks** | **8 weeks** |  |  |  |  |  |
| **Anticonvulsants (continued)** | | | | | | | | | | | | | | |
| Pregabilin | Lyrica |  |  |  |  |  |  |  |  | 300 |  | 600 |  |  |
| Semisodium Valproate | Depakote / Convulex |  |  |  |  |  |  |  |  | - |  | - |  |  |
| Sodium Valproate | Epilim |  |  |  |  |  |  |  |  | - |  | - |  |  |
| Tiagabine | Gabitril |  |  |  |  |  |  |  |  | 5 |  | 45 |  |  |
| Topiramate | Topamax |  |  |  |  |  |  |  |  | - |  | 300 |  |  |
| Zonisamide | Zonegran |  |  |  |  |  |  |  |  | 100 |  | 500 |  |  |
| **OPTIONAL SECTION D: OTHER REPORTED TREATMENTS FOR DEPRESSION AND RELATED MOOD DISORDERS^‡^** | | | | | | | | | | | | | | |
| Allopurinol | Zyloric |  |  |  |  |  |  |  |  | - |  | - |  |  |
| Amineptine |  |  |  |  |  |  |  |  |  | - |  | - |  |  |
| Armodafinil | Nuvigil |  |  |  |  |  |  |  |  |  |  | 150 |  |  |
| Atamoxetine | Strattera |  |  |  |  |  |  |  |  | 40 |  | 120 |  |  |
| Blonanserin | lonasen |  |  |  |  |  |  |  |  | - |  | - |  |  |
| Bromocriptine |  |  |  |  |  |  |  |  |  | - |  | - |  |  |
| Buprenorphine | Subutex / |  |  |  |  |  |  |  |  | - |  | - |  |  |
| Clonidine | Catapres / Dixarit |  |  |  |  |  |  |  |  | - |  | - |  |  |
| Clotiapine |  |  |  |  |  |  |  |  |  | - |  | - |  |  |
| Dexmethylphenidate | Focalin/ Attenade (US) |  |  |  |  |  |  |  |  | 5 |  | 30 |  |  |
| Etoperidone |  |  |  |  |  |  |  |  |  | - |  | - |  |  |
| Focalin/ Attenade (US) | Dexmethylphenidate |  |  |  |  |  |  |  |  | 5 |  | 30 |  |  |
| Hydroxyzine | Atarax / Ucerax |  |  |  |  |  |  |  |  | - |  | - |  |  |
| Indalpine |  |  |  |  |  |  |  |  |  | - |  | - |  |  |
| Inositol | Hexopal |  |  |  |  |  |  |  |  | - |  | 12000 |  |  |
| Ketanserin |  |  |  |  |  |  |  |  |  | - |  | - |  |  |
| Ketamine |  |  |  |  |  |  |  |  |  | 0.5mg/  kg IV |  | - |  |  |
| Melatonin |  |  |  |  |  |  |  |  |  | - |  | - |  |  |
| Metyrapone | Metopirone |  |  |  |  |  |  |  |  | 1000 |  | - |  |  |
| Mifepristone | Mifegyne |  |  |  |  |  |  |  |  | - |  | - |  |  |
| Mosopramine |  |  |  |  |  |  |  |  |  | - |  | - |  |  |
| Nimodipine | Nimotop |  |  |  |  |  |  |  |  | - |  | 180 |  |  |
|  |  |  |  |  |  |  |  |  |  |  |  |  |  |  |
| **Generic Name** | **Brand Names**  (UK, unless stated) | **Dose** | **Duration**  In weeks on a dose equal or greater that the minimum dose | **Tolerability & Response** | | | **Drug taken for at least:** | | | **Minimum Dose**  (mg/day unless stated) | **Equal or greater** | **Maximum**  **Dose***  (mg/day unless stated) | **Equal or greater** | **Augmentation** |
|  |  |  |  | **Tolerability^a^** | **Adherence^b^** | **Response^c^** | **4 weeks** | **6 weeks** | **8 weeks** |  |  |  |  |  |
| **Other reported treatments (continued)** | | | | | | | | | | | | | | |
| Nomifensine |  |  |  |  |  |  |  |  |  | - |  | - |  |  |
| Orphenadrine | Biorphen / Disipal |  |  |  |  |  |  |  |  | - |  | - |  |  |
| Pergolide |  |  |  |  |  |  |  |  |  | - |  | - |  |  |
| Perospirone | Lullan |  |  |  |  |  |  |  |  | - |  | - |  |  |
| Pipradrol |  |  |  |  |  |  |  |  |  | - |  | - |  |  |
| Prazosin | Hypovase |  |  |  |  |  |  |  |  | - |  | - |  |  |
| Propranolol | Inderal |  |  |  |  |  |  |  |  | - |  | - |  |  |
| Pyrovalerone |  |  |  |  |  |  |  |  |  | - |  | - |  |  |
| Rasagiline | Azilect |  |  |  |  |  |  |  |  | - |  | - |  |  |
| Remoxipride |  |  |  |  |  |  |  |  |  | - |  | - |  |  |
| Ritanserin | Tisterton (US) |  |  |  |  |  |  |  |  | - |  | - |  |  |
| Ropinirole | Adartrel / Requip |  |  |  |  |  |  |  |  | - |  | - |  |  |
| Rotigotine | Neupro |  |  |  |  |  |  |  |  | - |  | - |  |  |
| Seteperone |  |  |  |  |  |  |  |  |  | - |  | - |  |  |
| Setiptiline |  |  |  |  |  |  |  |  |  | - |  | - |  |  |
| St John’s Wort |  |  |  |  |  |  |  |  |  | - |  | - |  |  |
| Tamoxifen |  |  |  |  |  |  |  |  |  | 10 |  | 140 |  |  |
| Tandospirone |  |  |  |  |  |  |  |  |  | - |  | - |  |  |
| Teniloxazine |  |  |  |  |  |  |  |  |  | - |  | - |  |  |
| Thyroxine (T4) | Eltroxine |  |  |  |  |  |  |  |  | - |  | - |  |  |
| Tofenacin |  |  |  |  |  |  |  |  |  | - |  | - |  |  |
| Yohimbine |  |  |  |  |  |  |  |  |  | - |  | 30 |  |  |
| Zimelidine |  |  |  |  |  |  |  |  |  | - |  | - |  |  |

**Physical Treatments**

| ECT |  |  |  |  |  |  |  |  |  | - |  | - |  |  |
| --- | --- | --- | --- | --- | --- | --- | --- | --- | --- | --- | --- | --- | --- | --- |
| rTMS |  |  |  |  |  |  |  |  |  | - |  | - |  |  |
| VNS |  |  |  |  |  |  |  |  |  | - |  | - |  |  |
| Bright Light |  |  |  |  |  |  |  |  |  | - |  | - |  |  |
| tDCS |  |  |  |  |  |  |  |  |  | - |  | - |  |  |

*Notes:* The MTI is developed primarily for treatment resistant depression in “unipolar” depression. For purposes of the Maudsley Staging Method, treatments in Section A count towards antidepressant treatments and those in Section B towards augmentation treatments. Where two antidepressants are used in combination, count both as antidepressant trials under Section A. Although some combinations of antidepressants are more frequently used (e.g. venlafaxine plus mirtazapine, MAOI + TCA, SSRI + NRI) no specific weighting for these combinations is used for rating the Maudsley Staging Method. Section C (Other Antipsychotics/Mood Stabilisers) and Sections D (Other Reported Treatments For Mood Disorders) are optional and included for purposes of clinical utility. The list of drugs is deliberately inclusive so that all previous treatments can be assessed, and thus includes many that are not licenced or recommended for the treatment of depression; in addition, some have now been withdrawn in some countries. Inclusion does not indicate evidence of efficacy or a recommendation to prescribe. Prescribers should always check doses of treatments with which they are not familiar. *The MTI will be updated in line with the Maudsley Prescribing Guidelines to maintain evidence base for inclusion in Sections A and B, and for new additions to Sections C and D: please contact authors for updated versions*

*Dose ranges are taken from the following sources in order of preference: Maudsley Prescribing Guidelines, British National Formulary and manufacturer’s SPC. Dose ranges may refer to other indications where not well established for depression. Plasma levels are used for lithium in preference to dosage. Minimum or maximum undefined where left empty.

† - Included as first, second or third line augmentation strategies for TRD within Maudsley Prescribing Guidelines (Taylor et al 2015)

‡ - Not currently included in Maudsley Prescribing Guidelines; therefore, not scored for purposes of the Maudsley Staging Method

a – enter free text, or use Clinical Global Impression Treatment Related Adverse Effects (AEs) Index: 1, no AEs; 2, AEs do not significantly interfere with patient’s functioning; 3, AEs significantly interfere with patient’s functioning; 4, AEs outweigh therapeutic effect.

b – enter free text, or estimate percentage of doses that were taken. To be defined as an adequate trial adherence of at least 75% is needed.

c – enter free text, or use response based on Clinical Global Impression Scale: 1, very much improved; 2, much improved; 3, minimally improved; 4, no change; 5, minimally worse; 6, much worse; or 7, very much worse.

^H^ – dose in hospitalised patients; for consistency use the lower dose to determine whether maximum dose has been used

PO – oral; IM – intramuscular; IV – intravenous.

Physical treatment abbreviations: ECT – electroconvulsive therapy; rTMS – repetitive transcranial magnetic stimulation; VNS – vagus nerve stimulation; tDCS – transcranial direct current stimulation. Under dose and duration, a description of relevant parameters for each modality of treatment should be recorded, including where appropriate: bilateral v unilateral; frequency and total number of applications; intensity (e.g. electricity dose or length of seizure); and brain area targeted. No minimum criteria are stated due to current absence of sufficient evidence.

CGI scale based on Guy W, editor. ECDEU Assessment Manual for Psychopharmacology. 1976. Rockville, MD, U.S. Department of Health, Education, and Welfare
